# Characterizing Particle-Scale Acceleration of Mud-Pumping Ballast Bed of Heavy-Haul Railway Subjected to Maintenance Operations

**DOI:** 10.3390/s22166177

**Published:** 2022-08-18

**Authors:** Meng Wang, Yuanjie Xiao, Wenqi Li, Hongjun Zhao, Wenjun Hua, Yu Jiang

**Affiliations:** 1 School of Civil Engineering, Central South University, Changsha 410075, China; 2 Ministry of Education (MOE) Key Laboratory of Engineering Structures of Heavy Haul Railway, Central South University, Changsha 410075, China; 3 Chawu Track Division, Daqin Railway Co., Ltd., China Railway Taiyuan Group Co., Ltd., Beijing 101400, China

**Keywords:** heavy-haul railway, ballasted track, mud pumps, maintenance operations, tamping, particle-scale acceleration

## Abstract

Fouling and mud-pumping problems in ballasted track significantly degrade serviceability and jeopardize train operational safety. The phenomenological approaches for post hoc forensic investigation and remedies of mud pumps have relatively been well studied, but there still lacks studies on inherent mechanisms and ex ante approaches for early-age detection of mud pumps. This paper was aimed to exploring the feasibility of using particle acceleration responses to diagnose and identify early-age mud-pumping risks in real-world field applications. The innovative wireless sensors with 3D-printed shells resembling real shape of ballast particles were instrumented in the problematic railway section to monitor ballast particle movement prior to, during, and after maintenance operations, respectively. The real-time particle-scale acceleration data of ballast bed under both degraded and maintenance-restored clean conditions were recorded. The time histories, power spectra, and marginal spectra of 3D acceleration were comparatively analyzed. The results showed the 3D acceleration of ballast particles underneath rail-supporting tie plates displayed relatively clear periodicity of about 0.8 s with adjacent bogies regarded as a loading unit. The tamping operation was effective for compacting ballast bed laterally and improving the lateral interlocking of ballast particles, whereas the stabilizing operation was effective mainly in the lateral direction and for ballast particles underneath the sleepers. The mud pumps caused intensive particle-scale acceleration, and ballast particles underneath the sleepers were affected more severely than those in between adjacent sleepers. The ballast particles directly underneath tie plates exhibit dramatic acceleration variations due to maintenance operations as compared to those in other positions studied; hence, it seems promising to use particle-scale acceleration underneath tie plates as readily-implementable indicators for smart in-service track health monitoring.

## 1. Introduction

The ballasted track currently remains one of the few leading types of railway track structures due to the advantages in construction and maintenance [1,2]. However, the particulate nature of ballast particles often leads to performance degradation of ballasted trackbed. For example, the abrasion and breakage of ballast particles intensify with increasing axle load and train speed, thus causing the unfavorable densification, fouling, and clogging (i.e., reduced drainability) problems in ballasted tracked [3,4,5]; consequently, mud pumps, among other commonly observed track problems, can be prompted within such fouled ballasted trackbed [6,7]. Mud pumps could seriously degrade track stiffness and thus endanger operational safety of railway trains [8,9,10]. Traditional manual inspection and detection of mud pumps and other track problems are often labor-intensive, time-consuming, and subjective in nature; therefore, it becomes indispensable to develop automated, intelligent, and accurate means for the early-age diagnosis and detection of mud-pumping risks in ballasted trackbed so that remedial maintenance measures can be timely taken according to real-time health condition rather than the fixed schedules.

The root cause of mud-pumping fault has remained a widely-studied but challenging topic. Tadatoshi [11] proposed a suction-driven model and showed that the main cause of mud pumps is the intrusion of fine particles from the subgrade generated by the suction of ballast bed during the loading and unloading cycles. Raymond [12] found that the freeze-thaw cycles can cause fine-grained materials to pump out of the ballasted trackbed in winters according to a field performance investigation of the North American railway geotextiles. Duong et al. [13,14] believed that the interlayer materials between the subgrade and the ballasted trackbed were mainly generated by broken ballast particles, which then penetrated into the subgrade surface. The formerly Transportation Technology Center, Inc. (TTCI) established a field-testing zone to further study mud pumps [10,15,16,17]. Despite a considerable number of research studies have been carried out to explore the mechanisms of mud pumping fault, there still lacks radical countermeasures to prevent and control it in railway engineering practices.

The accurate early-age diagnosis and detection of mud pumps are the key step on which timely and effective prevention and control measures depend. The late-stage mud-pumping fault manifested on the surface of ballasted tracked is relatively easy to detect through routine labor-intensive methods; however, it is quite challenging to directly identify the early-age mud-pumping problem initiated inside the thick ballasted trackbed. The ground penetrating radar (GPR) technology has been widely applied in the non-destructive detection of structural faults in railway ballasted trackbed and subgrade [10,18,19,20]. Hugenschmidt [21] successfully applied GPR in the detection of railway subgrade problems for the first time in 1998. Since then, many countries including China have conducted related field and laboratory studies in this field [22,23]. Trong Vinh Duong et al. [13] carried out physical model tests and analyzed the influencing factors of the mud-pumping problem occurring in the interlayer between the ballast bed and underlying subgrade, including particle size distribution, moisture content, pore water pressure, hydraulic conductivity, etc. Kuo et al. [24] developed a characterized grid method and a scoring method to assess the mud-pumping distribution with an accuracy rate of 80%. Although the GPR technology has been reported to successfully detect visible or hidden mud-pumping problems in ballasted railway tracks [21], the accuracy and reliability of different GPR equipment and supporting post-processing software programs still vary considerably, not to mention the fact that they are highly costly and unaffordable for routine applications. In addition to GPR, other techniques have also been widely used for non-destructive detection of railway track foundation problems in recent years, such as the digital image correlation (DIC) [25,26,27], Interferometric Synthetic Aperture Radar (InSAR) [25], impact-echo method (IEM) [28], and synthetic aperture focusing technology (SAFT) [29,30]. However, these methods all require costly equipment and/or highly specialized skills that railway engineering practitioners usually lack. Therefore, to diagnose the in-service health condition and detect invisible problems of the ballasted trackbed accurately and reliably, it becomes imperative to explore automated, intelligent, and universally applicable methods in lieu of traditional ones.

The occurrence of mud pumps could cause uneven (or differential) rail track settlement and increasing track irregularities [31,32,33]. The existence of track irregularities could not only compromise the operational safety of heavy-haul trains but also degrade track substructures [34,35,36]. Li et al. [37,38] proposed a data-driven method for infrastructure deformation identification based on the characteristics of track geometry data, as well as a spatio-temporal identification model for identifying high-speed railway infrastructure deformation by using four years of track geometry data. Li et al. [39] analyzed the time and frequency characteristics of track geometry irregularity signals at the locations of mud pumps and used a multi-scale signal decomposition method to extract defect-sensitive features and then realize automatic detection of mud pumping problems. The nearly continuous and real-time track health monitoring of the entire rail networks could be possibly accomplished in a timely and cost-efficient manner by mounting robust sensors on in-service trains. For example, the problematic sections of railway track sub-structures were reportedly detected by using the vertical acceleration responses of a moving train [40]. Zeng et al. [41] proposed a data-driven approach for identifying mud pumps in the railway track substructure based on vibrational acceleration responses and Long Short-Term Memory (LSTM) artificial neural networks. The vibrational responses of ballastless slab tracks were also compared to detect the locations of mud pumps and study the feasibility of technical countermeasures to rectify and control the mud-pumping damage [42]. Therefore, analyzing the vibrational responses of the ballasted trackbed appears to be potentially helpful and promising for intelligent detection of mud-pumping problems in railway tracks.

Particle movement is a meso-scale manifestation of inter-particle contact behavior of ballast assemblies within the ballast bed; therefore, investigating the meso-scale movement characteristics of ballast particles may emerge as a promising, effective alternative to diagnose and identify the mud-pumping problem of ballasted tracked. The use of motion sensors (termed as “SmartRocks”) has been reported in the literature to directly capture real-time movement of ballast particles and then evaluate the field performance of ballasted trackbed under different in-service conditions [43,44,45,46,47]. The applications of such so-called SmartRock sensors in effective and accurate measurements of the vibrational responses of unbound aggregate particles including railroad ballasts were demonstrated in laboratory scaled model tests and triaxial tests [43,48,49,50]. Liu et al. [51] compared the ballast particle motion data measured by SmartRock sensors against those simulated by the discrete element method (DEM) model. Preliminary studies [52,53] suggested that SmartRock sensors could be used as a potential tool to quantify ballast behavior without using invasive measurement devices or disrupting railroad operations and to reflect the variations of dynamic behavior of ballasted trackbed under different substructure conditions. However, the widespread, reliable field applications of this new smart sensing technology for detecting invisible track defects such as mud pumps within ballasted trackbed remains to be extensively explored.

The purpose of this paper was to further study and substantiate the feasibility of SmartRock sensors in real-world field applications to diagnose and identify mud-pumping risks in ballasted trackbed. Therefore, a typical section of heavy-haul railway ballast bed with severe mud pumping problems was chosen for investigation, where the SmartRock sensors were employed and instrumented accordingly to monitor particle-scale acceleration responses prior to, during, and after major maintenance operations including ballast-cleaning and tamping. The three-dimensional (3D) acceleration responses and associated marginal spectra of ballast particles recorded by SmartRock sensors in different positions were comparatively analyzed for the initial degraded and subsequent rectified scenarios of the ballast bed. The findings are expected to contribute to the optimization of maintenance operation parameters and smart track health monitoring.

## 2. Field Test Section Studied and Instrumentation Plans

### 2.1. Description of Field Test Site and Railway Section Studied

**Figure 1:**
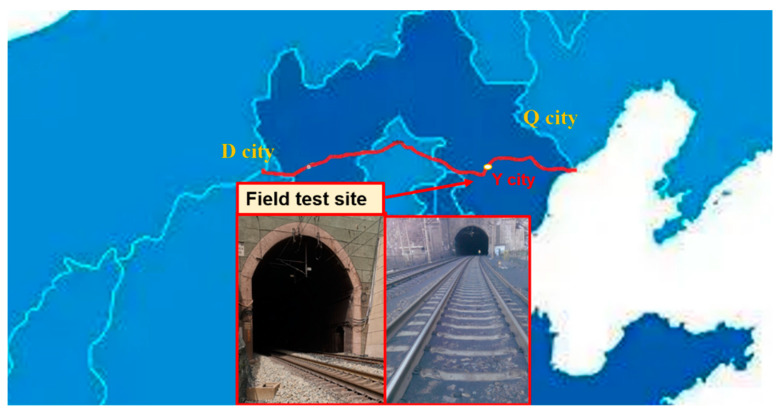
Location of field railway section studied.

**Figure 2:**
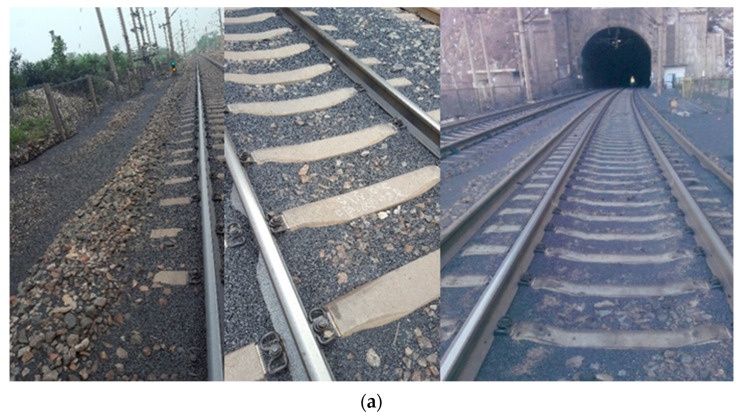

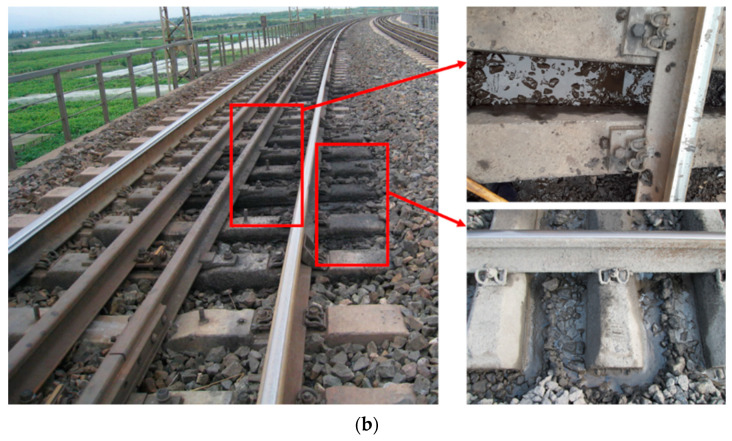
Illustration of the severity of typical ballast bed problems in the railway track section studied: (**a**) coal-dust fouling and (**b**) mud pumps.

It has to be declared herein that the identification information of the specific heavy-haul railway and geographical locations of the railway section studied are not disclosed in this paper upon the request of the related railway administration. The D railway, a flagship heavy-haul corridor in China dedicated exclusively to national coal transport, was shortlisted for this field study, as it features the fastest train running speed, the highest running density, the largest single railway volume, and the best transportation efficiency. Specifically, representative field positions in the railway section located in Y county (see Figure 1) were instrumented in this study. This railway section was claimed by the administration to suffer from repeated occurrences of track problems including coal dust fouling (see Figure 2a), mud pumps (see Figure 2b), and differential settlement. In this railway section, major maintenance operations are periodically scheduled to restore in-service health condition of the degraded ballast bed to its initial level as much as possible (e.g., fouling, densification, and drainage levels) and thus effectively mitigate track problems. The major types of in-service trains are H_XD1_ electric locomotives and C_80B_ wagons, of which the specific configuration parameters are listed in Table 1.

### 2.2. Description of SmartRock Sensors Used

**Figure 3:**
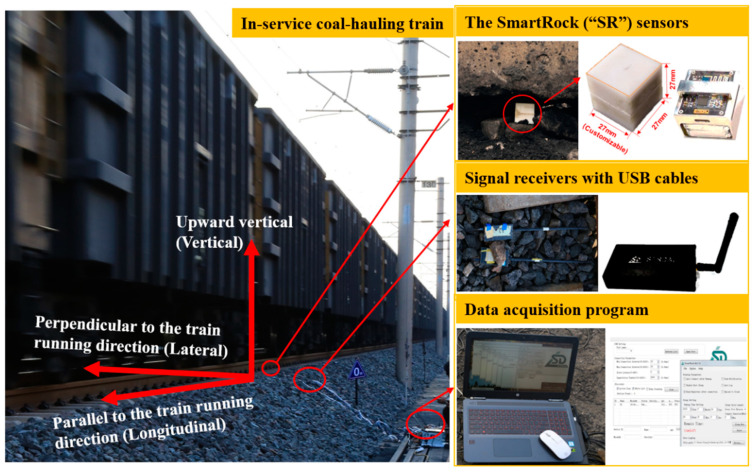
The SR sensors instrumented in the ballast bed of the railway section studied and key components of the corresponding data acquisition system.

The SmartRock (abbreviated as “SR” hereafter for brevity) sensors used in this study possess 3D printed cubic shells in which motion-sensing chips are encased, as shown in Figure 3. The 3D printed cubic shells are made of acrylonitrile butadiene styrene and nylon and resemble the hardness, toughness, and shape of typical granite ballast particles [44,45]. The geometric dimensions of the SR sensors are customizable upon specific demands. In this study, each of the SR sensors used measures a side length of 27 mm (i.e., the size of a typical ballast particle) and weighs 50 g, whereas its bulk density is close to that of regular ballast particles. The SR sensors used can directly record particle movement characteristics including translational acceleration, rotation angles, and contact forces in three dimensions. Since the morphology and surface texture of SR sensors are reasonably different from real ballast particles, the friction and interaction between individual SR sensors and real ballast particles are, strictly speaking, different from those merely among real ballast particles; however, such differences are too subtle to have significant influence on the movement characteristics of SR sensors, i.e., the movement of SR sensors can be regarded representative of that of real ballast particles. Therefore, the SR sensors were adopted and instrumented in the ballast bed of the chosen railway section to monitor the real-time ballast particle movement.

As shown in Figure 3, the signals of raw motion data recorded by SR sensors were transmitted wirelessly via Bluetooth to the receivers and then transferred via USB cables to the portable computer equipped with data acquisition software program. The receivers were placed near the shoulder of the ballast bed to ensure stable signal transmission during the data acquisition process. The sampling frequency of the signals can be adjusted through the accompanying data acquisition software program. In this study, the sampling frequency of transient motion measurements of each SR sensor was set as 100 Hz (i.e., 10 milliseconds per data point). Each SR sensor has a built-in local coordinate system in which its raw transient motion data were expressed. Prior to the data acquisition process, the initial position and orientation of each SR sensor instrumented were recorded for the purposes of the calibration and conversion between local and global coordinate systems, and their working status and data quality were checked carefully. To facilitate the comparisons of particle movement characteristics recorded in different positions inside the ballast bed, those SR sensors were instrumented with the same initial orientation in their built-in local coordinate system, i.e., the initial *x*-axis of SR sensors was perpendicular to the train running direction, the initial *y*-axis was parallel to the train running direction, and the initial *z*-axis was upward vertical.

**Figure 4:**
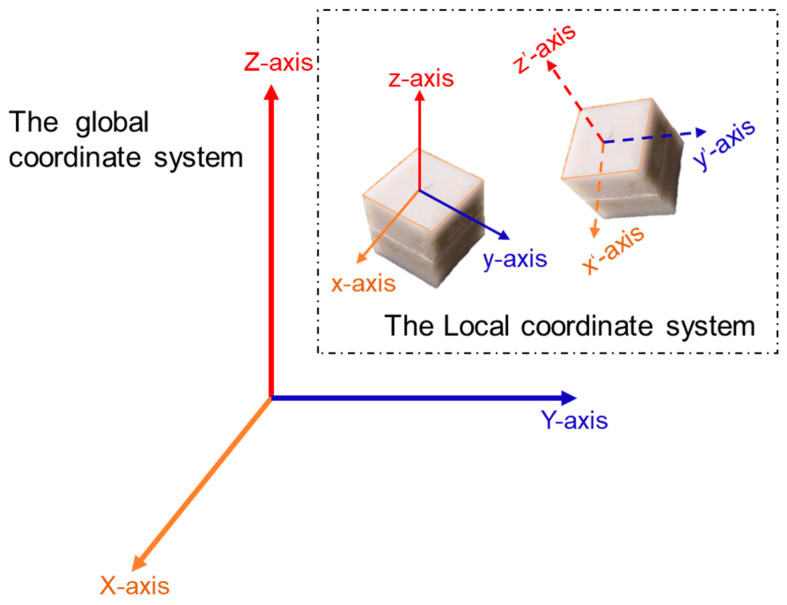
Illustration of the motion-dependent local coordinate system built in the SR sensors as relative to the fixed global coordinate system.

The raw particle-scale motion data collected by the instrumented SR sensors included three-dimensional (3D) acceleration and rotation responses expressed as quadratures in the constantly-changing, built-in local coordinate system at each time step [54]. Since the motion-recording chips are registered at the center of each SR sensor rather than at the center of its 3D printed shell, the acceleration data recorded by each SR sensor include not only the translational acceleration at the center but also the additional angular acceleration around the center (i.e., the motion of the fixed particle in the motion coordinate system). This fact should be considered accordingly. The acceleration and rotation in the local coordinate system (x, y, and z) can be converted to the global coordinate system (X, Y, and Z), as shown in Figure 4. To be specific, the inverse of the first quaternion was taken for all quaternions to solve for the quaternions in the global coordinate system and accomplish the conversions between two different coordinate systems; then, the raw acceleration data were multiplied by the inverse of the transformed quaternion to obtain the acceleration in the global coordinate system. This way, the real-time motion data of ballast particles collected under different spatial and temporal conditions can be cross-compared all in the same global coordinate system. Denote S = [p^x^; p^y^; p^z^; $p^{\dot{x}}$; $p^{\dot{y}}$; $p^{\dot{z}}$] where p^x^, p^y^, p^z^, $p^{\dot{x}}$, $p^{\dot{y}}$, and $p^{\dot{z}}$ represent the displacement and velocity components of the SR sensor in the global coordinate system, respectively, then denote s = [ω_x_; ω_y_; ω_z_] where ω_x_, ω_y_, and ω_z_ represent the angular velocity components of the SR sensor in the global coordinate system, respectively. Therefore, the motion measurement equations of the SR sensor at the k-th timestep can be described as follows:

(1) $$M_{k}=S_{K}+Z_{K}$$

(2) $$m_{k}=s_{K}+z_{K}$$

where *M_k_* is the vector of translational motion; *m_k_* is the vector of rotational motion; *Z_K_* is the vector of noise components in displacement and velocity measurements, and *z_K_* is the vector of noise components in angular velocity measurements.

### 2.3. Description of the Maintenance Operations

Two rounds of month-long comprehensive maintenance operations are enforced each year for the D railway infrastructure, one in April and the other in October. One of the maintenance goals is to effectively prevent, alleviate, or repair track problems including fouling and mud pumps and thus prolong the service life of ballasted track. Ballast-cleaning, tamping, and stabilizing are among the common track maintenance operations. The maintenance equipment consists of five railcars with each sequentially performing one of the five different operations: cleaning, tamping, re-tamping, leveling, and stabilizing. In this study, the acceleration responses of ballast particles were recorded during the processes of tamping, re-tamping, and stabilizing only, because the SR sensors buried in the ballast bed could be damaged or removed during the process of cleaning or leveling.

**Figure 5:**
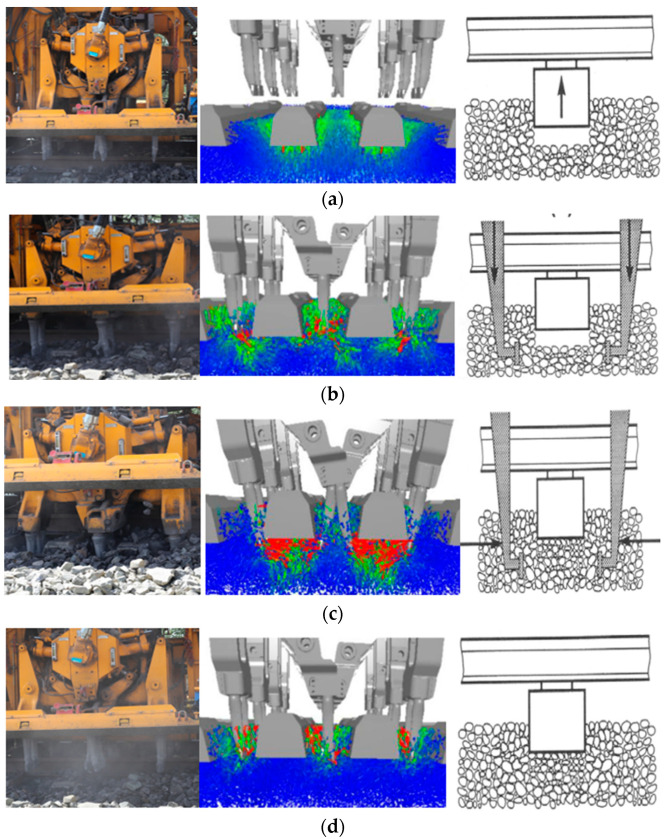
Illustration of the four stages of each cycle of tamping operation: (**a**) Sleeper-lifting stage; (**b**) Tine-inserting stage; (**c**) Ballast-squeezing stage; and (**d**) Tine-retracting stage.

The key step of the tamping operation is the regulated vibration of eight tamping tines inserted between every two adjacent sleepers [55]. The tamping process consists of four distinct, successive steps, namely sleeper-lifting stage, tine-inserting stage, ballast-squeezing stage, and tine-retracting stage [56,57,58], as shown in Figure 5. Note that the direction of the arrow in Figure 5a indicates the moving direction of the sleepers, and that the direction of the arrow in Figure 5b,c indicates the moving direction of the tines. In the sleeper-lifting stage, the sleepers were lifted to a pre-set height at a constant speed by applying a linear motion constraint. In the tine-inserting stage, the tamping tines were inserted into the ballast bed under the application of another linear motion constraint. Then, in the ballast-squeezing stage, the tamping tines were rotated to squeeze the ballast particles underneath the sleepers and were controlled by a rotating motion constraint. Finally, in the tine-retracting stage, the rotation direction of tamping tines was opposite to that in the ballast-squeezing stage, and the tamping tines were pulled out upward. In addition, a sinusoidal vibration constraint was applied to the tamping tines throughout the whole tamping process with a vibration frequency of 35 Hz, thus generating impact forces to compact the ballast bed.

### 2.4. Field Instrumentation Plans

**Figure 6:**
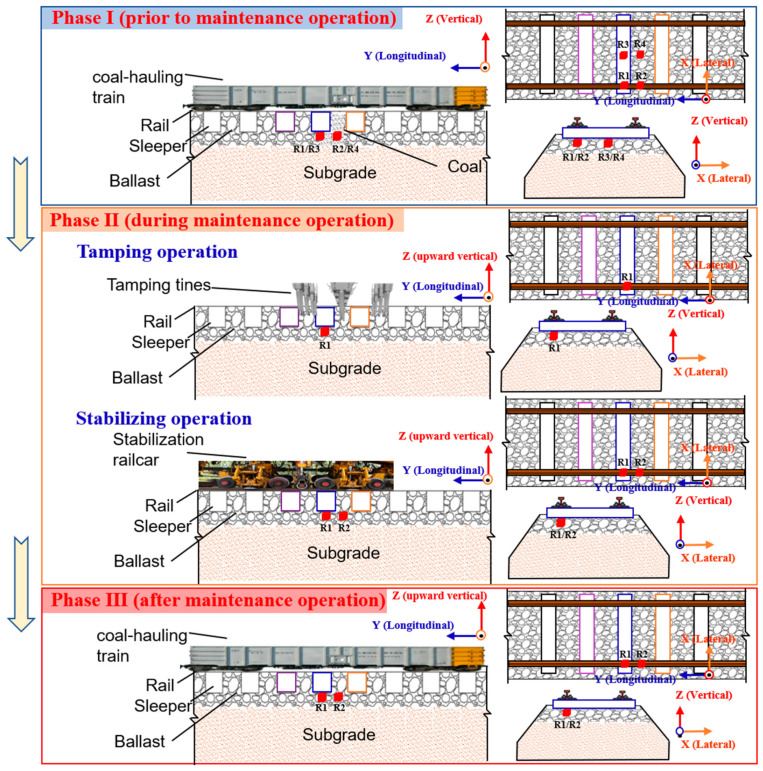
Schematic diagram of the locations of instrumented SR sensors in the ballast bed during three different phases of the field test.

In the chosen section of the D railway, mud-pumping problems are repeatedly observed near a tunnel exit where coal dusts blown off from the coal-hauling wagons severely accumulate at the top surface of the ballast bed (see Figure 2). To collect the particle-scale acceleration responses, four SR sensors were instrumented in different positions of the ballast bed prior to maintenance operations, and two SR sensors were placed in the pre-determined positions during and after maintenance operations (mainly ballast-cleaning, tamping, and stabilizing), respectively, as shown in Figure 6.

**Figure 7:**
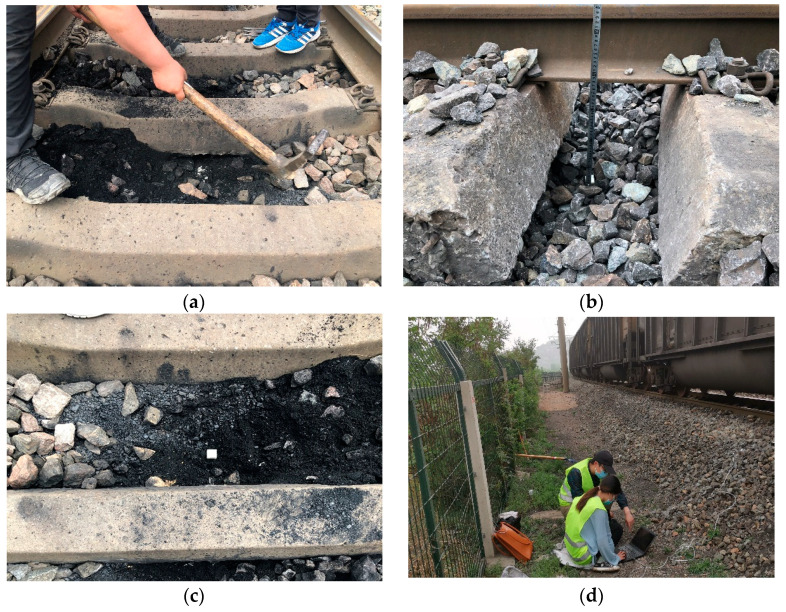
Field Installation sequences of the SR sensors: (**a**) Instrumentation excavation; (**b**) Sensor placement; (**c**) Ballast backfill; (**d**) Data acquisition.

During phase I of the field test, four SR sensors were placed in the pre-determined positions of the ballast bed prior to maintenance operations (where typical track problems including mud pumps were observed), i.e., immediately underneath the center of the rail-supporting tie plate (#R1), in the center of two adjacent tie plates (#R2), immediately underneath the center of the sleeper (#R3), and in the center of two adjacent sleepers (#R4), as shown in Figure 6. Note that such four SR sensors were placed at the same depth level from track surface. The goal was to record real-time ballast particle acceleration responses and provide promising particle-scale data support for disclosing the inherent mechanisms of mud-pumping problem. During phase II of the field test, two SR sensors were placed in the pre-determined positions (at the same depth level) of the ballast bed during tamping operations, i.e., immediately underneath the center of the rail-supporting tie plate (#R1) and in the center of two adjacent tie plates (#R2), as shown in Figure 6. The goal was to record real-time ballast particle acceleration responses for assessing the tamping-induced compaction quality of the ballast bed. Finally, during phase III of the field test, two SR sensors were placed in the pre-determined positions (at the same depth level) of the ballast bed after maintenance operations to compare the particle-scale acceleration responses between two different track health conditions (i.e., mud-pumping versus clean). The specific field instrumentation process of those SR sensors in the ballast bed was photographed in Figure 7. As presented previously, the sampling frequency of transient motion measurements of such SR sensors was set as 100 Hz.

## 3. Methodology of Particle-Scale Acceleration Data Analysis

**Figure 8:**
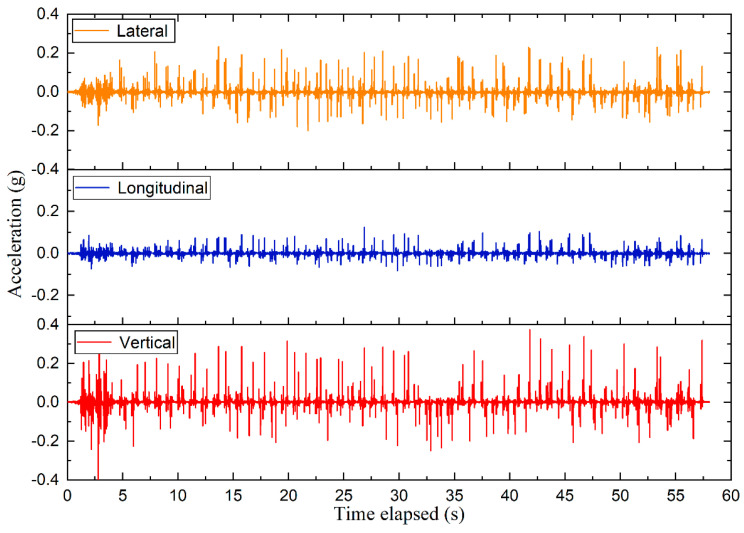
The time-history curves of 3D acceleration responses recorded by #R2 in the ballast bed and normalized for zero initial values.

The time-history curves can well reflect the variations of 3D acceleration responses of ballast particles with time. As shown in Figure 8, the 3D acceleration responses recorded by #R2 (located immediately underneath the center of the rail-supporting tie plate) prior to the maintenance operation were taken as examples to evaluate the acceleration variations of ballast particles with time. Note that the initial value of the vertical acceleration is −1*g due to the existence of the acceleration of gravity (g). To facilitate comparisons of 3D acceleration responses, the vertical acceleration data were first normalized by zeroing the initial values, thus making the initial acceleration values in three dimensions all zero.

**Figure 9:**
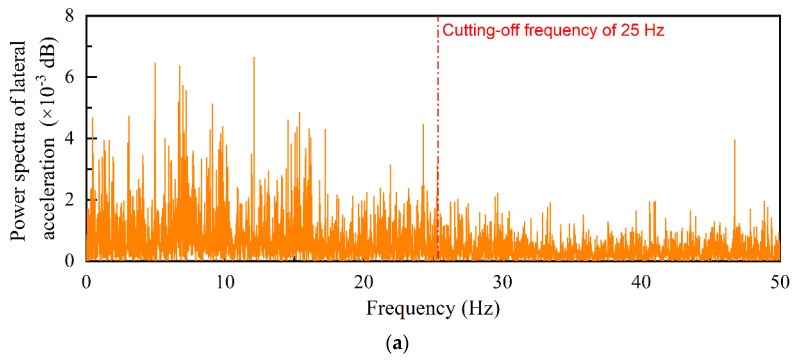

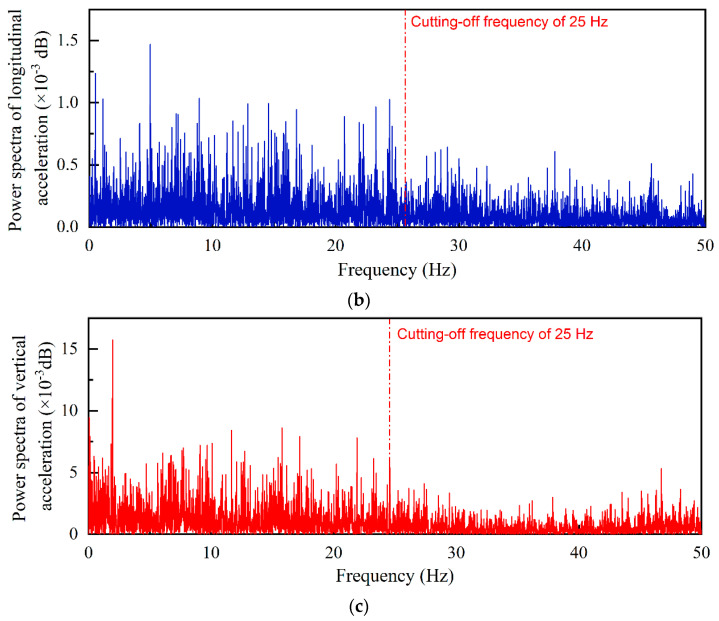
Examples of the power spectra of normalized acceleration data recorded by #R2 in three different dimensions: (**a**) Lateral, (**b**) Longitudinal, and (**c**) Vertical.

**Figure 10:**
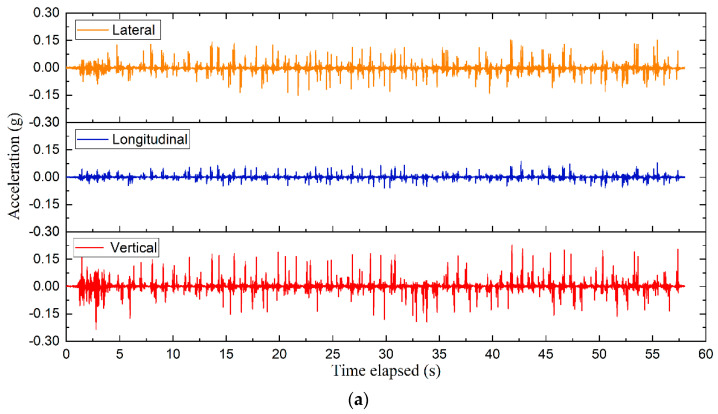

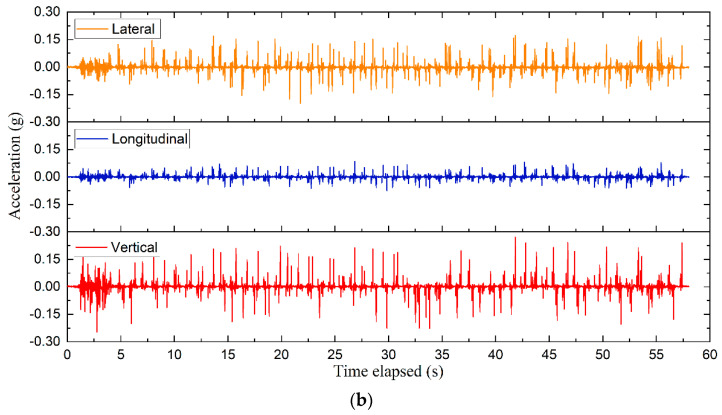
Comparison of the time-history acceleration data processed by (**a**) the IIR low-pass and (**b**) the FFT low-pass filtering methods.

The acceleration data normalized for zero initial values were further processed to effectively filter and remove environmental noise. Determining the cut-off frequency is the key to low-pass filtering. Therefore, the power spectra of the normalized acceleration data in three dimensions were obtained by the fast Fourier transform (FFT), of which the examples are illustrated in Figure 9. It shows that the noise components caused by the surrounding environment are complicated, and that the dominant frequencies of the 3D acceleration responses are slightly different. The frequency range of 0–25 Hz is observed to possess the greatest power in all three different dimensions. Therefore, the infinite-impulse-response (IIR) low-pass filters and the fast-Fourier-transform (FFT) low-pass filters were then applied separately to the 3D acceleration data by using the cutting-off frequency of 25 Hz. The example results are shown in Figure 10a and Figure 10b, respectively.

It can be seen from Figure 10 that the time-history curves of the filtered acceleration data are quite similar to those of the initial acceleration data, and that almost no data distortion is observed for the low-pass filtering. To compare the data fidelity of the IIR and FFT low-pass filtering methods, the Pearson correlation coefficient values and corresponding statistical significance levels among the filtered and initial acceleration data were calculated with the results summarized in Table 2. It can be seen from Table 2 that the acceleration data filtered by both low-pass filtering methods with the same cutting-off frequency of 25 Hz are significantly correlated with the initial acceleration data. The correlation coefficient values are greater for the acceleration data processed by the FFT low-pass filtering, whereas the correlation coefficient values for the lateral acceleration data are lower than those for the longitudinal and vertical acceleration data. This indicates that the environmental noise is more considerable in the lateral direction. Therefore, the FFT low-pass filtering method was selected for use in this study to process the field-measured acceleration data.

**Figure 11:**
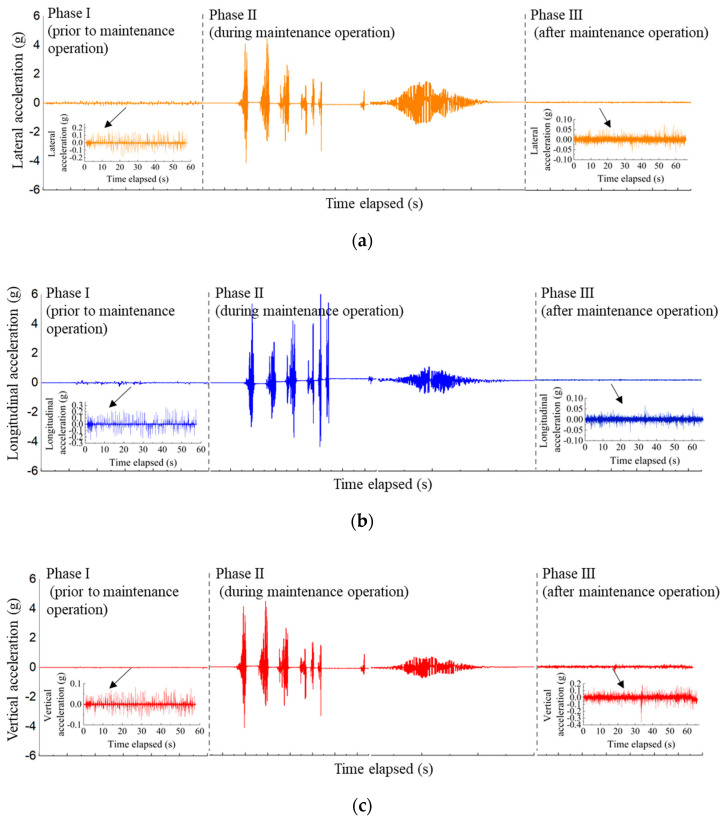
The time-history curves of processed (**a**) lateral, (**b**) longitudinal, and (**c**) vertical acceleration responses recorded by #R1 prior to, during, and after maintenance operations.

By following the above-described data preprocessing procedures, the time-history curves of processed 3D acceleration responses recorded by the SR sensor #R1 during three different phases of the field test (i.e., prior to, during, and after maintenance operations, respectively) were shown as examples in Figure 11. The detailed analysis results of the whole data collected in the field test are presented in the subsequent sections.

## 4. Results and Analysis

### 4.1. Tamping-Induced Particle-Scale Acceleration Responses

**Figure 12:**
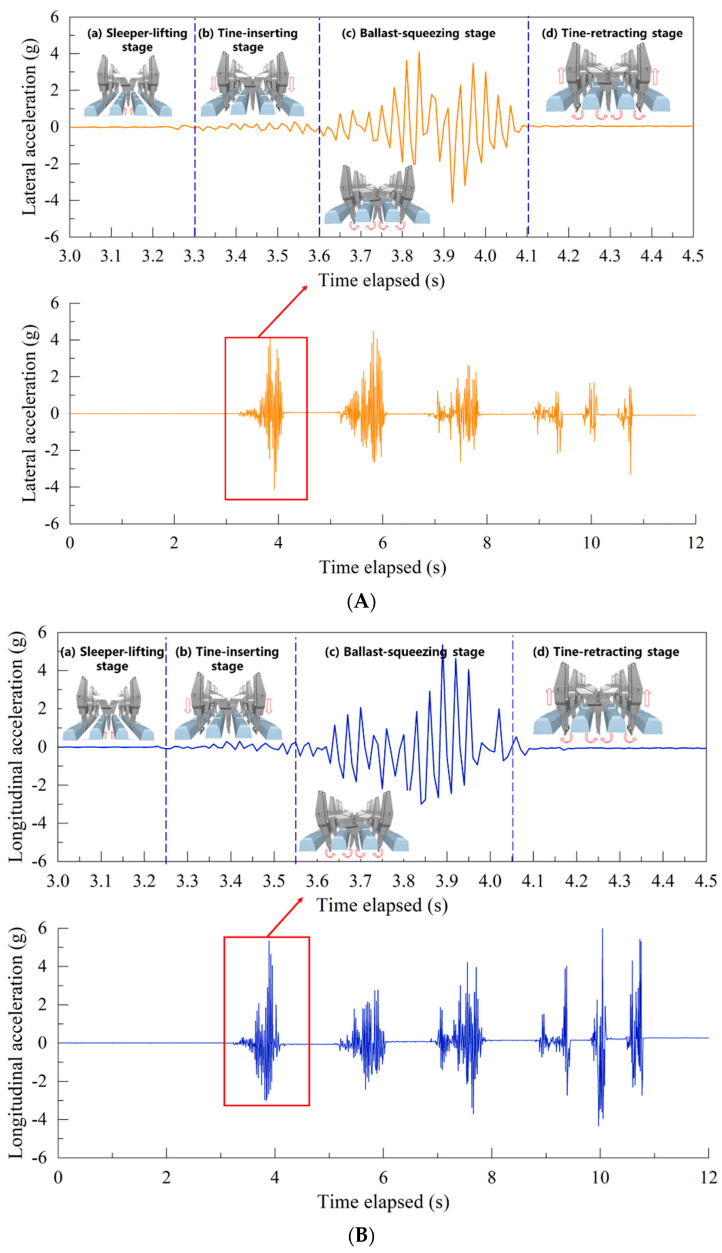

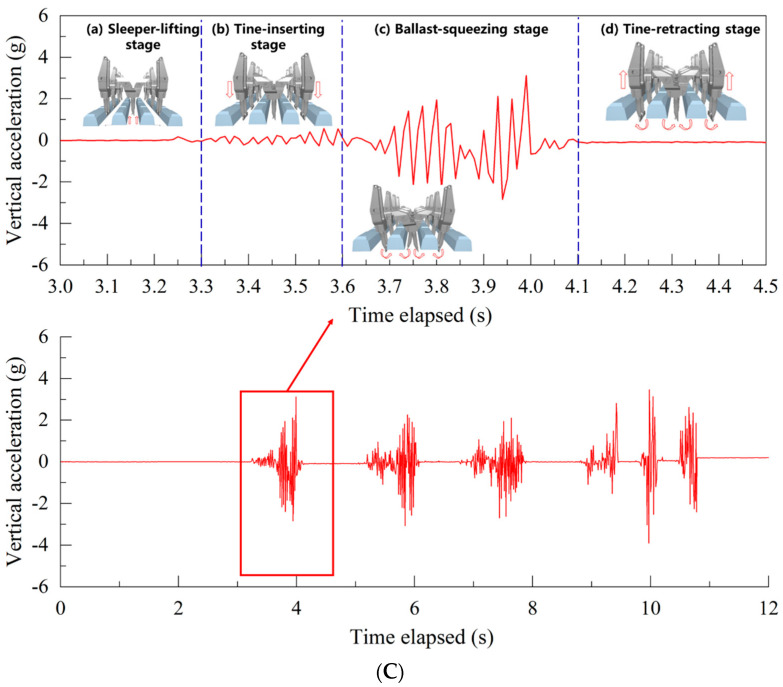
The time-history curves of (**A**) lateral, (**B**) longitudinal, and (**C**) vertical acceleration responses recorded by #R1 during tamping operation.

The key step of the tamping operation is the regulated vibration of eight tamping tines inserted between every two adjacent sleepers. It is worth noting that in order to prevent the SR sensors from being damaged by the tamping tines, they were placed only in positions underneath the center of the rail-supporting tie plate (i.e., denoted as #R1 in Figure 6). To further analyze the particle-scale acceleration responses of ballast particles in the above-mentioned four stages of each tamping cycle, the first tamping cycle was selected and illustrated as an example in Figure 12. It can be seen from Figure 12 that with the up-lifting of the sleepers and the insertion of the tamping tines, the ballast particles barely move. During the tine-inserting stage, the acceleration amplitudes of the ballast particles start to increase gradually, and the ballast particles start to move slightly due to the insertion of the tamping tines. Both sleeper-lifting and tine-inserting stages last for about 0.3 s (s). In the ballast-squeezing stage (i.e., about from 3.6 s to 4.1 s), the acceleration amplitudes of the ballast particles reach the maximum, and the ballast particles move dramatically due to the vibration and rotation of the tamping tines. In the tine-retracting stage, as the tamping tines retract from and exit the ballast bed, ballast particles cease to move. The 3D acceleration responses all exhibit consistent trends, thus revealing, from the perspective of particle-scale movement, the effectiveness and inherent mechanism of the tamping operation.

The time-history curves of 3D acceleration responses recorded by the SR sensor #R1 during the tamping operation are shown in Figure 12. It can be seen that almost no vibration or particle movement is observed when the tamping tines were not inserted into the ballast bed. With the insertion of tamping tines into the ballast bed, the acceleration amplitudes in all three dimensions appear to increase rapidly. The acceleration amplitudes then decreased rapidly to near zero as the tamping tines departed from the sensor location. As such, it can be clearly identified from the variations of acceleration amplitudes that the ballast bed was tamped by six cycles. The longitudinal acceleration amplitudes are the greatest as compared to vertical and lateral ones, because the clamping forces exerted by tamping tines and the vibration of tamping tines are both longitudinal in nature during the tamping operation. The lateral acceleration amplitudes gradually decrease as the number of tamping cycles increases, thus proving that tamping operation is effective to compact the ballast bed laterally and improve the lateral interlocking of ballast particles. The longitudinal acceleration amplitudes remain approximately unchanged during the tamping operations due to the vibration effect of tamping parameters. The vertical acceleration amplitudes are relatively low, further confirming that the vibration of the tamping tines is mainly horizontal in nature.

**Figure 13:**
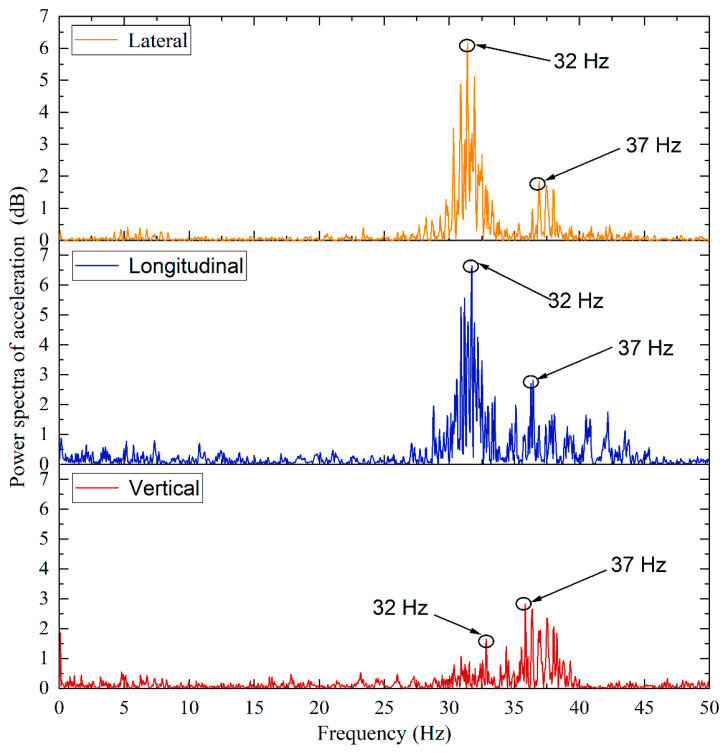
The calculated power spectra of 3D acceleration responses recorded by #R1 during tamping operation.

To analyze the vibration characteristics of the tamping tines during the tamping operation, the power spectra of 3D acceleration responses are calculated and drawn in Figure 13. The frequency distribution curves of both lateral and longitudinal acceleration responses concentrate in the range of 30–35 Hz. This confirms that the lateral and longitudinal vibration frequencies of the tamping tines are almost identical. However, the power spectra of vertical acceleration responses within the frequency range of 30–35 Hz are relatively low, indicating that the horizontal vibration of the tamping tines is predominant. In contrast, the highest power spectra of vertical acceleration responses are observed within the frequency range of 35–40 Hz, which is mainly attributable to the vertical vibration of the tamping railcars.

**Figure 14:**
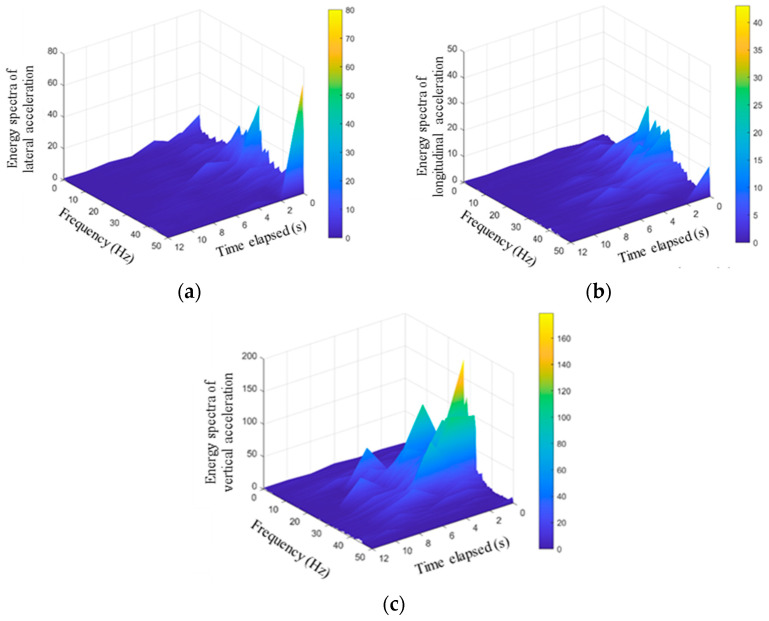
The calculated Hilbert spectra of (**a**) lateral, (**b**) longitudinal, and (**c**) vertical acceleration responses recorded by #R1 during tamping operation.

The high-resolution time-frequency analysis can be realized by the Hilbert spectra, and the variations of 3D acceleration amplitudes with both time and frequency over the entire frequency range can then be visualized. The original 3D acceleration responses recorded by the SR sensor #R1 during the tamping operation were subjected to the empirical modal analysis (EMD), where 13 intrinsic mode function (IMF) components were obtained, and the Hilbert-Huang transform (HHT) analysis was performed on all components. The calculated Hilbert spectra of 3D acceleration responses recorded by the SR sensor #R1 are presented in Figure 14. The Hilbert spectra color-code the magnitudes of energy with the color closer to yellow representing greater energy magnitude. It can be seen from Figure 14 that the energy magnitudes of lateral acceleration responses are the greatest within the dominant frequency range of 25–35 Hz. The Hilbert energy spectra of longitudinal acceleration responses are relatively greater in the first 6 s, which corresponds to the second cycle of tamping operation. Similarly, the Hilbert energy spectra of vertical acceleration responses are relatively greater in the first 8 s, corresponding to the completion of the first three cycles of tamping operation. The Hilbert energy spectra of lateral acceleration responses are relatively greater in the first 10 s, which corresponds to the fifth cycle of tamping operation. Therefore, the comprehensive comparison of the Hilbert energy spectra of 3D acceleration responses indicates that the effective number of tamping cycles is approximately 4. The desired level of compaction of the ballast bed studied may be achieved by applying 4 cycles of tamping operation.

### 4.2. Stabilizing-Induced Particle-Scale Acceleration Responses

During the stabilizing operation (following tamping operation), the wheels of stabilizing railcars vibrate laterally, and the vibration energy is then transmitted down into the ballast bed to further increase post-tamping stability of the ballasted trackbed. Since no mechanical equipment is intruded into the ballast bed during the stabilization step, no damage to the SR sensors would occur. Therefore, the SR sensors were placed in two different positions denoted as #R1 and #R2 (see Figure 6). The sampling frequency of the SR sensors was also set to 100 Hz for data acquisition during the stabilizing operation.

**Figure 15:**
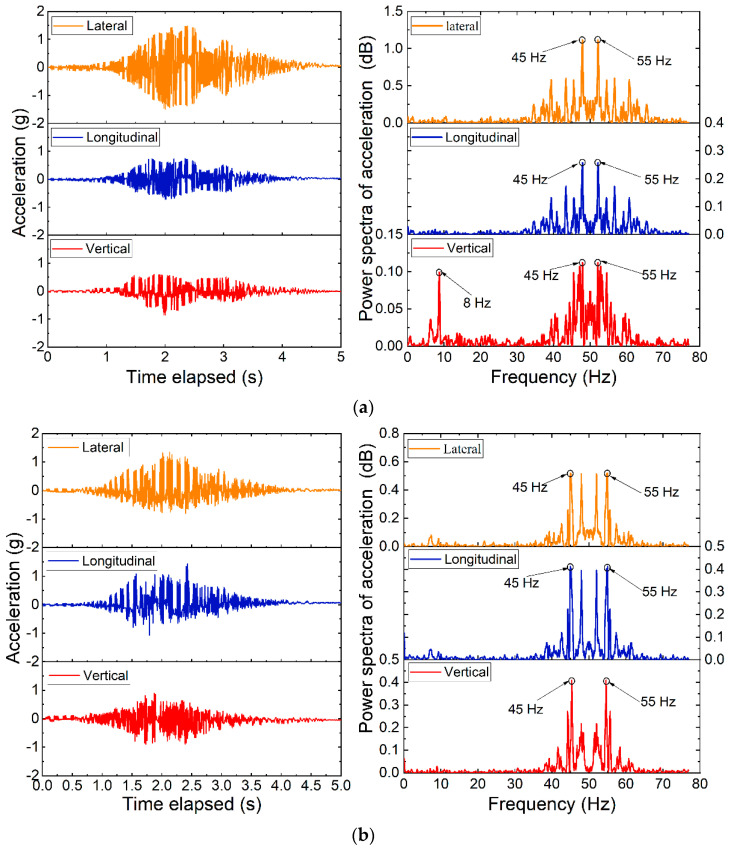
The time-history curves and calculated power spectra of 3D acceleration responses recorded by (**a**) #R1 and (**b**) #R2 during stabilization steps of tamping operation.

The time-history curves of 3D acceleration responses recorded in such two positions during the stabilizing operation are plotted in Figure 15. It can be clearly seen from Figure 15 that the lateral acceleration amplitudes are greater than those in the vertical and longitudinal directions. This is consistent with the vibration direction of the vibrating wheels of the stabilization railcars. Hence, the degree of compaction of the ballasted trackbed achieved during stabilization steps can be reflected by the vibration-induced acceleration responses recorded by the SR sensors. The lateral acceleration amplitudes recorded by the SR sensor #R1 are greater than those by #R2. This confirms that the vibration responses of ballast particles underneath the sleepers during the stabilization steps are more intensive than those in between the sleepers. The longitudinal vibration responses of ballast particles are also observed due to the horizontal extrusion among them. The ballast particles underneath the sleepers are directly affected by the stabilization operation as compared to those in between the sleepers. Note that the vertical acceleration responses of ballast particles are mainly generated by dynamic loading of the stabilizing railcars. As for both lateral and longitudinal acceleration responses recorded by the SR sensor #R2, their positive amplitudes are greater than negative ones. This indicates that ballast particles near this position may move unidirectionally probably due to the relatively weak horizontal stability. This may further imply that the horizontal stability of the ballast shoulder needs to be strengthened.

As can be seen from the calculated power spectra of 3D acceleration responses recorded by the SR sensors #R1 and #R2 in Figure 15, similar power spectra distributions are observed for both lateral and longitudinal acceleration responses, which are however relatively different from those of the vertical acceleration responses. Such differences in power spectra distributions indicate that the predominant impact of the stabilization operation is concentrated horizontally. The power spectra of lateral acceleration responses are significantly greater than those of longitudinal and vertical acceleration responses recorded in the same position, whereas the power spectra of lateral acceleration responses recorded by the SR sensor #R1 are greater than those by #R2. This further confirms that the stabilization operation is effective mainly in the lateral direction and for ballast particles underneath the sleepers. However, the power spectra of longitudinal and vertical acceleration responses recorded by the SR sensor #R1 are lower than those by #R2, which indicates that the longitudinal and vertical vibration responses of ballast particles in between the sleepers are more intensive due to their relatively lower degree of compaction and thus interlocking.

### 4.3. Comparison of Particle-Scale Acceleration Responses before and after Maintenance Operations

The fouling materials in the ballast bed, which are mainly coal dusts for the D railway, are removed massively by the specialized ballast-cleaning machine during the track maintenance operations. The fouling level of the ballasted trackbed can thus be reduced dramatically. To compare the 3D acceleration responses of ballast particles under the mud-pumping versus clean conditions of the ballasted trackbed, the acceleration data recorded by the SR sensors #R1 and #R2 (see Figure 6) were analyzed. Note that the sampling frequency of both sensors was set as 100 Hz during the data acquisition process.

**Figure 16:**
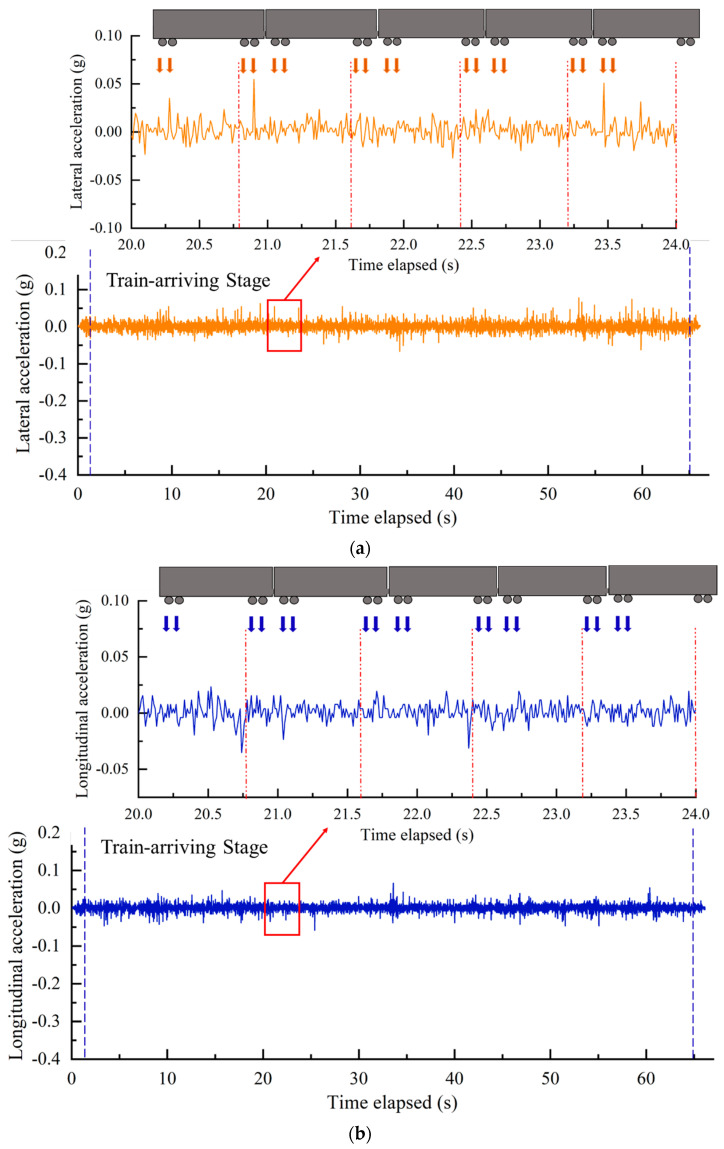

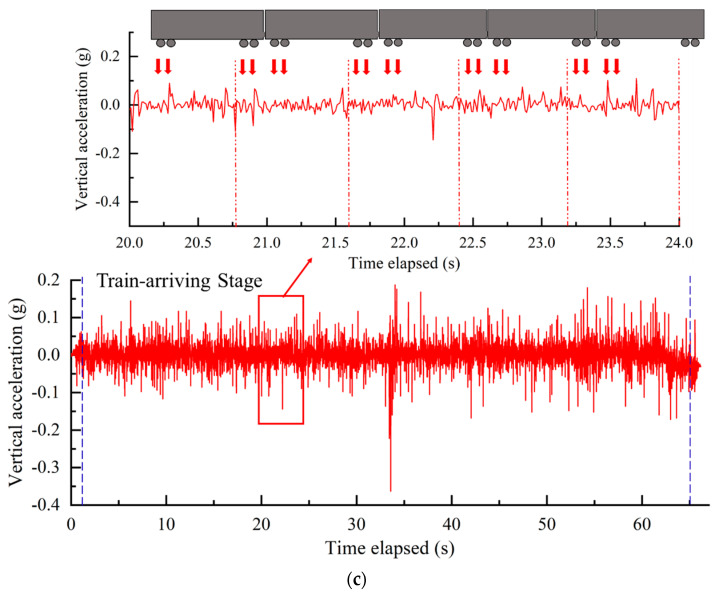
The time-history curves of (**a**) lateral, (**b**) longitudinal, and (**c**) vertical acceleration responses recorded by #R1 after maintenance operations.

The time-history curves of 3D acceleration responses recorded by the SR sensors #R1 are plotted in Figure 16. As can be seen from Figure 16, when the railcars pass by the sensor locations continuously, the acceleration amplitudes of ballast particles underneath the sleepers fluctuate within a certain range under the combined application of external cyclic wheel loading and internal excitations such as inter-particle contact forces. The vertical acceleration responses of ballast particles in the clean ballast bed increases significantly at approximately 33 s when the heavier locomotive passes by this position. The acceleration responses in three dimensions overall exhibit clear periodicity with three distinct, successive stages (i.e., train-approaching, train-arriving, and train-departing). During the train-approaching stage, the time-history curves of acceleration are close to 0, i.e., the heavy-haul train hadn’t arrived at the instrumentation locations yet. During the subsequent train-arriving and train-departing stages, the time-history curves of acceleration feature gradually-increasing and then decreasing amplitudes as the heavy-haul trains approached and then departed the instrumentation locations, respectively. The acceleration amplitudes in three different dimensions descend in the following order: vertical > lateral > longitudinal. The acceleration in the vertical direction is significantly (about 4–5 times) greater than that in the other two directions. This indicates that the lateral track stability at the instrumented railway section is relatively weak. All the acceleration amplitudes are observed to be relatively small, as the D railway track was constructed with high standards and remains relatively stable after continuous busy service. In addition, the acceleration responses of the ballast particles underneath the rail-supporting tie plates showed a certain periodicity, which is about 0.8 s corresponding to the cyclic loading frequency of about 1.25 Hz. This observation can be subsequently used to determine a reasonable loading scheme for laboratory model tests and triaxial tests of ballast materials.

**Figure 17:**
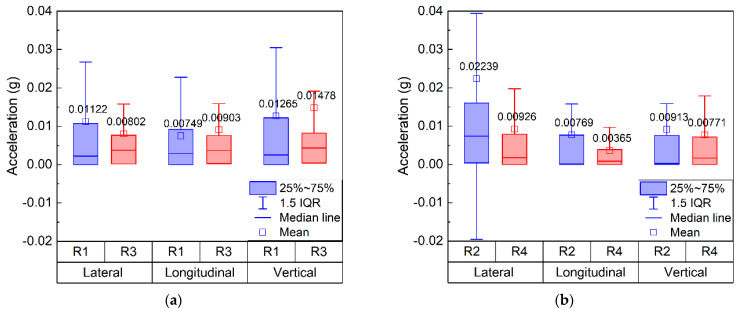
The box plots showing statistical distributions of 3D acceleration recorded (**a**) below the sleepers and (**b**) in-between the two adjacent sleepers at the same depth of the ballast bed prior to maintenance operations.

After the raw acceleration data recorded by SR sensors in different positions prior to maintenance operations were processed by the FFT low-pass filtering method to remove noise interference, they were further subjected to statistical analysis. It is worth noting that acceleration responses have positive or negative signs depending on their directions, therefore their absolute values were used for subsequent statistical analysis and comparisons. The example box plots are provided in Figure 17 where the SR sensors are grouped laterally (i.e., #R1 vs. #R3 and #R2 vs. #R4). The box-shaped statistical distribution diagrams can intuitively reflect the extreme values and the overall distribution characteristics of 3D acceleration data. Note that the SR sensors #R1 and #R3 were embedded at the same depth immediately below the sleepers, whereas the SR sensors #R2 and #R4 were embedded at the same depth in between two adjacent sleepers.

As shown in Figure 17, the average values of vertical acceleration recorded by SR sensors #R1 and #R3 are greater than their lateral and longitudinal counterparts, but the average values of lateral acceleration recorded by #R2 and #R4 are greater than their longitudinal and vertical counterparts. This indicates that the lateral restraint in between the two adjacent sleepers is relatively insufficient and the lateral track stability near the ballast shoulder may need to be properly strengthened. The average acceleration responses recorded by #R1 are significantly lower than those by #R3, but the average acceleration responses recorded by #R2 are significantly greater than those by #R4, indicating that the detrimental influence of the mud pumping problem on the vibrational acceleration responses of ballast particles under the sleepers is more significant than that in between the two adjacent sleepers.

Note that the SR sensors #R1 and #R2 were embedded at the same depth below the identical rail beam, whereas the SR sensors #R3 and #R4 were embedded at the same depth in between the two rail beams. The average values of lateral acceleration recorded by #R1 and #R2 are greater than their longitudinal counterparts, which is explained by the fact that the lateral track stability at such two positions is relatively weaker than the longitudinal track stability. This implies that the lateral track stability near the ballast shoulder may need to be properly strengthened. Similarly, the average values of lateral acceleration recorded by #R4 are greater than their longitudinal counterparts as well. The average acceleration responses recorded by #R3 are significantly greater than those by #R4, which is expected as greater train loading is transmitted onto the former locations. According to the load-transferring principle, the average values of lateral acceleration recorded by #R2 should be lower than those by #R1; however, the opposite trends are actually observed in Figure 17. This may imply the lateral restraint in the position of #R2 is more insufficient than that in the position of #R1.

**Figure 18:**
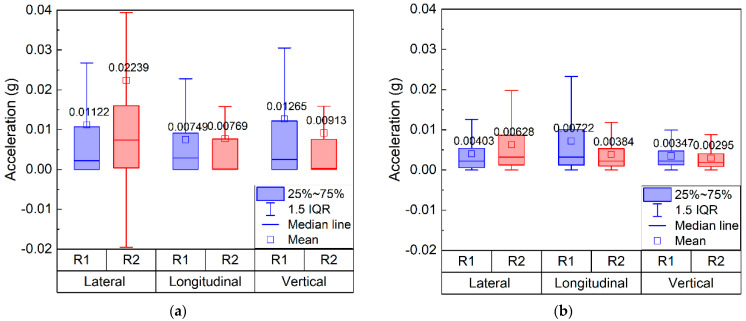
The box plots showing statistical distributions of 3D acceleration recorded by #R1 and #R2 at the same depth of the ballast bed (**a**) prior to and (**b**) after maintenance operations.

The statistical distributions of 3D acceleration responses recorded by SR sensors #R1 and #R2 at the same depth of the ballast bed prior to and after the maintenance operations are comparatively shown in Figure 18. The average values of vertical acceleration recorded by #R1 and #R2 are the greatest both prior to and after maintenance operations. The average values of lateral acceleration recorded by #R1 and #R2 are greater than their longitudinal counterparts both prior to and after maintenance operations. The average values of longitudinal and vertical acceleration recorded by #R1 are greater than those recorded by #R2 prior to and after maintenance operations, which is expected as greater train loading is transmitted onto the former locations. However, the average values of lateral acceleration recorded by #R1 is lower than those recorded by #R2 prior to and after maintenance operations, implying that the lateral restraint in the position of #R2 is more insufficient than #R1. The 3D acceleration responses (especially vertical acceleration) recorded by #R1 and #R2 prior to maintenance operations are much greater than those after maintenance operations, thus confirming the improvement of in-service health condition of the ballast bed.

As shown in Equation (3), the Empirical mode decomposition (EMD) was performed to obtain the marginal spectrum distributions of 3D acceleration responses recorded by the SR sensors in the mud-pumping susceptible ballast bed before and after maintenance operations, respectively. The marginal spectra of 3D acceleration data were calculated to quantify the vibrational energy distribution within each frequency band in the form of energy spectra. Seven main modal functions extracted are transformed. The energy spectra of the vibrational acceleration data in the frequency-amplitude scale can be obtained by integrating Equation (3), as shown in Equation (4).

(3) $$E\left(\omega ,t\right)=Re\sum_{i=1}^{n+1}a_{i}\left(t\right)\cdot e^{j\int \omega_{i}\left(t\right)dt}$$

(4) $$e\left(\omega ,t\right)=\int_{-\infty }^{\infty }E\left(\omega ,t\right)\;dt$$

where *E*(*ω*,*t*) is the energy spectra of the vibrational acceleration data, $a_{i}\left(t\right)$ is the amplitude of the vibrational acceleration, n is the number of vibration acceleration data points collected, and *e*(*ω*,*t*) is the marginal spectra of the vibrational acceleration data.

**Figure 19:**
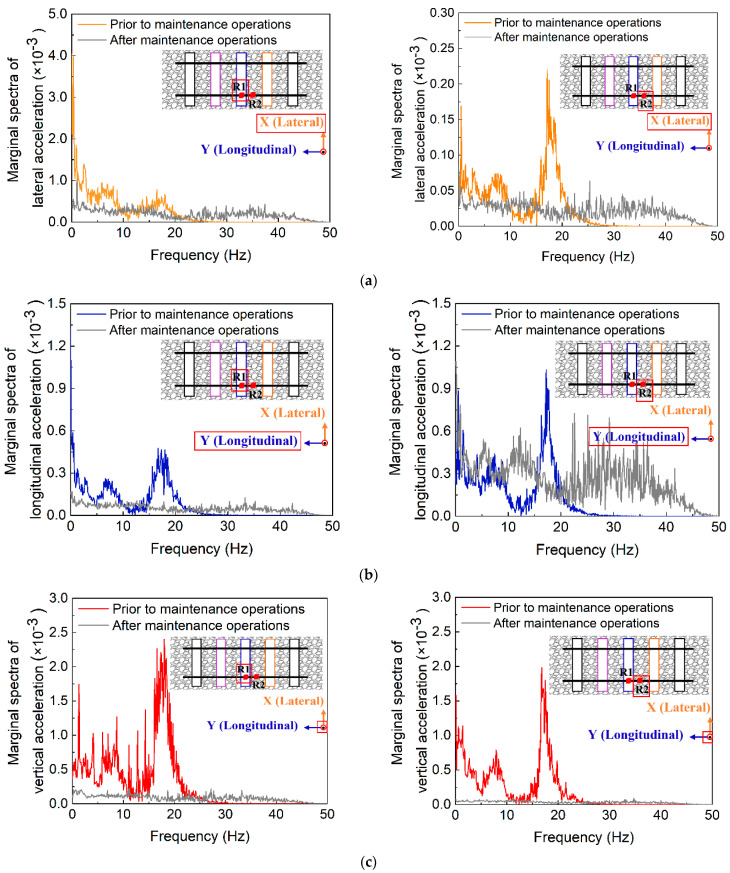
Comparison of marginal spectra of (**a**) lateral, (**b**) longitudinal, and (**c**) vertical acceleration responses prior to and after maintenance operations.

The comparison results are visualized in Figure 19. Note that R1 and R2 denote the SmartRock sensors placed in the related positions denoted by the red bullets. It can be seen from Figure 19 that the marginal spectra of vertical acceleration responses are the greatest, but the smoothness levels of corresponding marginal spectrum distribution curves vary significantly among different positions in the ballast bed. In addition, the marginal spectra of 3D acceleration responses recorded before the maintenance operations including ballast-cleaning and tamping are greater than those after the maintenance operations, whereas the corresponding marginal spectrum distribution curves before the maintenance operations are much less smooth than those after the maintenance operations. This indicates that the existence of mud pumping problem in the ballast bed could cause considerable vibration responses of ballast particles, which consequently accelerates ballast degradation and leads to differential track settlement. The ballast particles located directly underneath the center of rail-supporting tie plate (i.e., #R1) exhibit more dramatic variations in acceleration responses before and after the maintenance operations. It thus seems promising to use the real-time movement characteristics of ballast particles underneath the tie plates as intelligent, non-destructive, and readily-implementable indicators for monitoring and evaluating the in-service health condition of ballasted trackbed.

## 5. Summary and Conclusions

In this study, the 3D (i.e., lateral, longitudinal, and vertical) acceleration responses of ballast particles in the mud-pumping susceptible ballast bed of a flagship heavy-haul railway were monitored by the innovative SmartRock (“SR”) sensors. The acceleration data recorded in different positions during three distinct phases (i.e., prior to, during, and after maintenance operations) of the field test were analyzed and compared. The major conclusions can be drawn as follows.

- Among the four distinct, successive steps of each cycle of tamping operation (i.e., the sleeper-lifting, tine-inserting, ballast-squeezing, and tine-retracting stages), the ballast-squeezing stage plays a key role. During this stage, the acceleration amplitudes of ballast particles reach the maximum, and ballast particles move dramatically due to the vibration and rotation of tamping tines. The lateral acceleration amplitudes gradually decrease as the number of tamping cycles increases, thus confirming that tamping operation is effective for compacting the ballast bed laterally and improving the lateral interlocking of ballast particles. The ballast bed studied can achieve a relatively dense condition with about 4 cycles of tamping operation.
- The degree of compaction of the ballast bed achieved during stabilization steps can be reflected by the vibration-induced acceleration responses recorded by the SR sensors. The power spectra of lateral acceleration responses are significantly greater than those of longitudinal and vertical counterparts recorded in the same position. The stabilization operation is effective mainly in the lateral direction and for ballast particles underneath the sleepers.
- The lateral stability of the ballasted trackbed studied is relatively weaker than the longitudinal stability. This implies that the lateral track stability near the ballast shoulder may need to be properly strengthened. The detrimental influence of the mud-pumping problem on the vibrational acceleration responses of ballast particles under the sleepers is more significant than that in between two adjacent sleepers. The mud-pumping problem significantly reduces the inter-particle interlocking of the ballast bed, and the resulting intensive particle movement as reflected by dramatic acceleration responses leads to uneven settlement of the ballasted trackbed.
- The ballast particles located directly underneath the center of rail-supporting tie plates exhibit more dramatic variations in acceleration responses prior to and after the maintenance operations. It thus seems promising to use the real-time movement characteristics of ballast particles underneath the tie plates as intelligent, non-destructive, and readily-implementable indicators for monitoring and assessing the in-service health condition of ballasted trackbed.
- The 3D acceleration responses of ballast particles underneath the rail-supporting tie plates show a relatively clear periodicity, which is about 0.8 s corresponding to the cyclic loading frequency of about 1.25 Hz. This observation can be subsequently used to determine a reasonable loading scheme for laboratory model tests and triaxial tests of ballast materials.

In order to further validate the findings made in this study, more extensive field studies are needed by using the SR sensors and are currently underway. The results of 3D acceleration responses of ballast particles in different positions of the ballast bed studied can be useful for the calibration and comparison of related discrete element method (DEM) models to be developed for disclosing inherent micromechanical mechanisms of track problems.

## Figures and Tables

**Table 1:** Configuration parameters of typical railcars operated in the D railway.

Railcar Type	Axle Load (Metric Tons)	Wheelbase Interval (mm)	Interval of Adjacent Wheelbases (mm)	Length between Bogie Centers (mm)	Railcar Length (mm)
H_XD1_	25	2800	5800	9000	17,600
C_80B_	25	1830	1970	8200	12,000

**Table 2:** Comparison of statistical correlations between original acceleration data and those processed by each of the IIR and FFT low-pass filtering methods.

Filtering Method	Statistics	X Direction	Y Direction	Z Direction
IIR	Correlation coefficient	0.784	0.804	0.803
	Significance level	Significant	Significant	Significant
FFT	Correlation coefficient	0.808	0.835	0.833
	Significance level	Significant	Significant	Significant

## Data Availability

Some or all data, models, or code that support the findings of this study are available from the corresponding author upon reasonable request.
